# Associations of Birth Interval With Prevalence of Depression in Postmenopausal Women

**DOI:** 10.1155/da/8066072

**Published:** 2025-04-15

**Authors:** Ronghua Zuo, Yan Chen, Qiaoying Zhu, Yu Liu, Shuang Yao, Qinmin Bao, Dake Li

**Affiliations:** ^1^Department of Anesthesiology, Peking University Third Hospital, No. 49, North Garden Street, Haidian District, Beijing 100191, China; ^2^Department of Neurology, The Affiliated Jiangning Hospital to Nanjing Medical University, No.169, Hushan Road, Jiangning District, Nanjing, Jiangsu 211100, China; ^3^Department of Gynecology, Women's Hospital of Nanjing Medical University (Nanjing Women and Children's Healthcare Hospital), Nanjing, Jiangsu 210018, China

**Keywords:** age at first birth, age at last birth, birth interval, depression, postmenopausal women

## Abstract

**Background:** This study aims to explore the relationship between birth interval and prevalence of depression among postmenopausal women with two deliveries in the United States.

**Methods:** Data from the National Health and Nutrition Examination Survey (NHANES) were used, which spanned the years 2005–2018 and is publicly accessible. We utilized weighted multivariable logistic regression analysis, restricted cubic splines (RCS), and subgroup analysis to examine the relationship between the prevalence of depression in postmenopausal women with only two deliveries and the age at first birth (AFB), age at last birth (ALB), and birth interval (the difference between ALB and AFB).

**Results:** A total of 2375 postmenopausal women with only two deliveries were included in the study, and 271 (11.4%) had depression. RCS models showed that AFB and ALB were U-shaped curves associated with the prevalence of depression. Additionally, the birth interval was roughly L-shaped curve correlated with the risk of depression.

**Conclusions:** Both early and late childbearing, as well as short birth intervals, may contribute to mental health challenges in this demographic. These findings suggest that women with both early and late childbearing, as well as those with short birth intervals, may face a higher risk of depression during their postmenopausal years. This underscores the importance of targeted mental health screening and support for these groups.

## 1. Introduction

Depression is a significant public health concern, affecting millions of individuals worldwide [1, 2]. Among postmenopausal women, the prevalence and risk factors for depression are of particular interest due to the complex interplay of hormonal, physiological, and psychosocial factors [3, 4]. However, sex-specific factors such as the timing of reproductive events, which directly impact a woman's hormonal trajectory throughout her life, were largely understudied [5, 6]. Despite the known associations between reproductive history and mental health, research often overlooks the nuanced ways in which specific reproductive events impact depression risk in later life [5]. Understanding how these sex-specific factors interact with hormonal changes can shed light on the biological underpinnings of depression in postmenopausal women.

Reproductive history, including age at first birth (AFB), age at last birth (ALB), and birth interval, is a significant factor in a woman's life course [7]. Guo et al. [8] concluded that an earlier AFB is significantly associated with an increased risk of frailty among middle-aged and older women. An earlier AFB was also significantly associated with an increased risk of lung cancer, particularly lung adenocarcinoma, within subpopulations characterized by a higher genetic susceptibility and the presence of adverse behavioral risk factors [9]. Reproductive history may play a crucial role in the development of depression later in life. Previous studies have extensively explored the association between AFB and depression, yielding inconsistent results [10–12]. Some research indicates that early childbearing may increase the risk of depression [13, 14]. For instance, Mirowsky and Ross [13] found that teenage mothers were at higher risk for depression later in life. Also, a Mendelian randomization study confirmed a causal relationship, suggesting that later AFB may reduce the risk of postpartum depression (PPD) [15, 16]. However, the existing literature has primarily focused on depression during reproductive years or immediately postpartum, with limited attention to the long-term effects on postmenopausal women. Furthermore, while the link between AFB and depression has been extensively studied, there is a notable research gap concerning the associations of ALB and birth interval with depression risk. This gap is particularly significant given that birth interval encompasses both AFB and ALB, potentially providing a more comprehensive picture of a woman's reproductive history and its long-term mental health implications [17, 18]. As women live longer and spend a greater proportion of their lives in the postmenopausal state, understanding these relationships becomes increasingly important for public health and clinical practice. This study aims to address these research gaps by exploring the associations between AFB, ALB, birth interval, and the prevalence of depression among postmenopausal women in the United States. By utilizing data from the National Health and Nutrition Examination Survey (NHANES), we seek to elucidate these relationships and provide insights that may inform clinical practice and public health strategies for depression prevention and management in postmenopausal women.

## 2. Materials and Methods

### 2.1. Study Population

**Figure 1:**
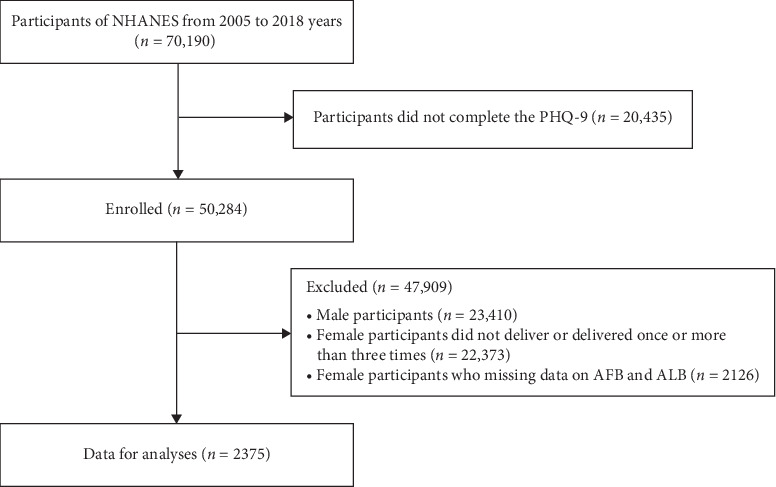
Study flow chart. NHANES, National Health and Nutrition Examination Surveys.

All data are from the NHANES data from 2005 to 2018. The survey, organized by the National Center for Health Statistics (NCHS) and the Centers for Disease Control and Prevention, is a continuous, systematic collection and analysis of health-related data. A total of 70,190 individuals were included, and 20,435 participants were excluded due to a lack of Patient Health Questionnaire Depression Self-Rating Scale (PHQ-9) questionnaires. Additionally, we excluded 23,410 men and eliminated 22,373 females who did not deliver or only delivered once or more than three times. Furthermore, 2126 females without reproductive factors (AFB and ALB) were also excluded. Finally, 2375 postmenopausal women with two deliveries only were included in the analysis (Figure 1). The NCHS Ethics Review Board approved the protocol and obtained written informed consent from all participants.

### 2.2. Reproductive Factors

The Reproductive Health Questionnaire was used to assess reproductive factors in women. Researchers used the questionnaire to collect information on a woman's AFB, ALB, and number of deliveries. Birth interval was defined as the years between ALB and AFB. The researchers also collected participants' age at menarche, number of pregnancies, age at menopause, fertile lifespan (difference between age at menarche and age at menopause), history of gynecological surgery (including oophorectomy and hysterectomy), oral contraceptive use, and female hormone use.

### 2.3. Covariates

The covariates included in this study were age, race/ethnicity, education level, family income to poverty ratio (PIR), blood urea nitrogen (BUN), hypertension, estimated glomerular filtration rate (eGFR), marital status, alcohol user, triglycerides (TG), smoker, waist circumference (WC), hyperlipidemia, diabetes mellitus (DM), congestive heart failure (CHF), angina pectoris, coronary heart disease (CHD), heart attack and stroke, body mass index (BMI), mean energy intake, uric acid (UA), fast blood glucose (FBG), serum creatinine (Scr), total cholesterol (TC), and high-density lipoprotein-cholesterol (HDL) [19–23]. Detailed covariate information can be obtained from the NHANES database (https://www.cdc.gov/nchs/nhanes/index.htm).

### 2.4. Depression Ascertainment

The PHQ-9, a commonly utilized depression screening instrument in clinical settings, was used to evaluate depression diagnoses. The PHQ-9 provides four response possibilities for each question: 0 = not at all, 1 = a few days, 2 = more than half the days, and 3 = almost every day. The scale has a total score of 27 points, and when the total score is >10, the participant is judged to have major depressive disorder.

### 2.5. Statistical Analysis

Statistical analysis was conducted using R Version 4.2.3 and SPSS 23.0. A *p*-value of less than 0.05 was deemed statistically significant. The “survey” package was used for sample weight computation [19]. Continuous variables are described using mean ± standard deviation (SD), while categorical variables are represented by frequency (%). The Weighted Student's *t*-test and chi-square test were employed to assess between-group differences for continuous and categorical variables. Weighted multivariable logistic regression analysis examined the relationship between AFB, ALB, and birth interval with depression. Model 1 was adjusted for age and race/ethnicity, while model 2 was adjusted for age, race/ethnicity, education level, smoking, alcohol use, marital status, family poverty-to-income ratio, hypertension, and DM. Model 3 was developed from model 2, including adjustments for the CHD, heart attack, CHF, angina pectoris, stroke, hyperlipidemia, CKD, oral contraceptive use, female hormone use, age at menarche, hysterectomy, fertile lifespan, bilateral oophorectomy, BMI, WC, mean energy intake, UA, Scr, BUN, TC, eGFR, and HDL. Furthermore, restricted cubic splines (RCS) and subgroup analysis were employed to examine the relationship between AFB, ALB, and birth interval with depression. Subgroup analyses were conducted to investigate the potential modifications in the effects of AFB, ALB, and birth interval on depression, influenced by factors such as race/ethnicity, education level, family PIR, hysterectomy, female hormone use, and bilateral oophorectomy, as per model 3.

## 3. Results

### 3.1. Baseline Characteristics

Table 1 presents the baseline characteristics of the study population. The prevalence of depression among postmenopausal women was 11.4% (271/2357). The group exhibiting depressive symptoms was significantly younger than the group without depressive symptoms (*p*=0.002). Furthermore, AFB, ALB, birth interval, age at menarche, age at menopause, and reproductive lifespan were all significantly decreased in individuals exhibiting depressive symptoms compared to those without depression (*p*<0.001).

### 3.2. Association of Age at First Birth, Age at Last Birth, and Birth Interval With Depression

**Figure 2:**
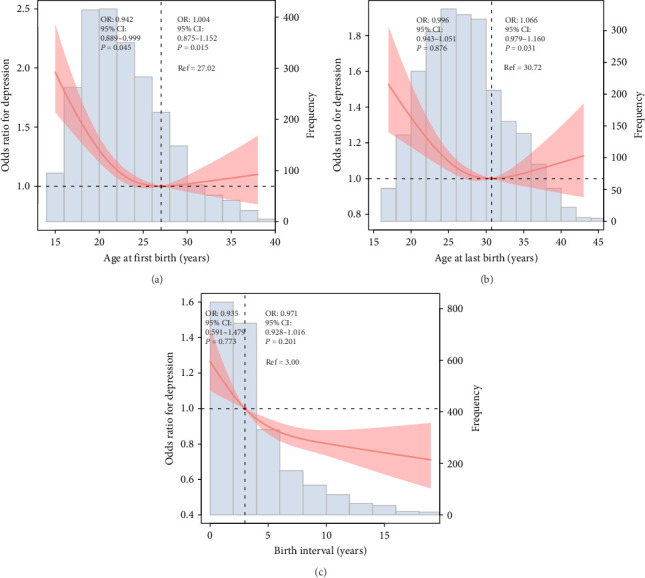
Restricted cubic spline plots of associations between (A) age at first birth, (B) age at last birth, (C) birth interval, and the prevalence of depression in postmenopausal women.

The RCS model was employed to illustrate the variation in depression risk associated with increasing AFB, ALB, and birth interval, following the adjustment for covariates. The results of the RCS curve showed that AFB and ALB were U-shaped curves related to the risk of depression (AFB, *p* for nonlinearity = 0.015; ALB, *p* for nonlinearity = 0.031; Figure 2A,B). Additionally, birth interval was roughly L-shaped curve associated with depression risk (*P* for nonlinearity = 0.201, Figure 2C). Additionally, the association of AFB, ALB, and birth interval with risk of depression is presented in Table 2.

### 3.3. Subgroup Analyses

Subgroup analyses stratified by race/ethnicity, education level, family PIR, hysterectomy, female hormone use, and both ovaries removed were further performed (Supporting Information Figures S1–S3; Supporting Information Tables S1–S3). The U-shaped curve association of AFB with depression was found among participants who were other Hispanic, other race, family PIR ≥1.3, or more than high school education groups, as well as in those without hysterotomy, bilateral oophorectomy, or oral contraceptive use (Supporting Information Figure S1). Additionally, there was a U-shaped curve correlation between ALB and depression found in other Hispanic, nonHispanic Black, nonHispanic White, and family PIR <1.3 or ≥1.3 groups, as well as in those without hysterotomy or bilateral oophorectomy (Supporting Information Figure S2). Finally, we also found that the roughly L-shaped curve association of birth interval with depression was observed in Mexican American participants, those with a family PIR ≥1.3, more than a high school education, or a history of hysterotomy, bilateral oophorectomy, or no oral contraceptive use (Supporting Information Figure S3).

## 4. Discussion

Our study revealed that the associations of AFB and ALB with the risk of depression exhibited U-shaped curves. Both exceptionally early and late initial and final parturitions were associated with an elevated risk of depression among postmenopausal women. Furthermore, postmenopausal women with two deliveries who experienced shorter birth intervals between their first and second parturitions demonstrated a higher propensity for depressive symptoms. These findings suggest that the temporal aspects of childbearing may exert enduring effects on women's psychological well-being, in which extremes in reproductive timing, whether precocious or delayed, may heighten susceptibility to depressive symptoms in later life.

Our findings on U-shaped associations between ALB and AFB with the risk of depression among postmenopausal women in the US indicate that the AFB and ALB often coincide with significant life transitions that impact mental health. Aligned with previous research [10, 11], the associations between AFB and the risk of depression among postmenopausal women are nonlinear, with both very early (teenage) and very late AFB (after age 35) linked to increased depression risk. Early childbirth can alter life trajectories, influencing educational and career opportunities, which in turn affect mental health. Furthermore, later AFB may be linked to PPD, potentially leading to long-term mental health implications [15, 16]. Similarly, the relationship between ALB and depression risk is also complex, with both very early and very late ALB potentially increasing the risk of depression. Early ALB may limit educational and career opportunities, while late ALB may pose challenges related to more complications and older parenting [24–26]. For example, women who have children later in life may face the stress of caregiving in tandem with the onset of menopause, potentially increasing the risk of depression [26].

Interestingly, the roughly L-shaped relationship between birth interval and depression risk suggests that women with shorter intervals between their two births may be at higher risk for depression. Biological and ecological reasons could explain these findings. Biologically, the duration of a woman's reproductive span is associated with cumulative hormonal exposure [27–29]. A longer or shorter reproductive span might influence the onset and severity of postmenopausal symptoms, including depression [5]. For instance, an extended reproductive span may be associated with prolonged exposure to estrogen, which has protective effects against depression [28]. Socially, this could be due to various factors, including increased stress from closely spaced pregnancies, limited time for physical and emotional recovery between births, or potential financial strains associated with raising two children in quick succession [30, 31]. Women who experience a shorter gap between their first and last births might experience more intense caregiving responsibilities in a compressed timeframe [5, 31]. Social expectations and role overload can exacerbate stress and depression, particularly in the postmenopausal period when social support networks may change [6].

Our research contributes to the growing body of evidence linking reproductive factors to mental health outcomes in women [5, 6]. It emphasizes the need for a life-course perspective in understanding and addressing women's mental health, particularly as they transition through menopause and beyond [6]. Moreover, exploring sex-specific factors like AFB, ALB, and their differences is essential for understanding the complex interplay between biological, psychosocial, and environmental factors in the development of depression among postmenopausal women [5]. Despite its strengths, including a large, nationally representative sample and comprehensive statistical analysis, the study has limitations. The cross-sectional design restricts causal inferences, and the reliance on self-reported data may introduce recall bias [32]. In this study, we used multivariable logistic regression, RCS, and subgroup analysis to assess the association between birth intervals and depression in postmenopausal women. Future research should expand the sample size, compare American and Chinese populations, and explore advanced methods like machine learning to further investigate these relationships. Furthermore, the focus on postmenopausal women with two deliveries limits the generalizability of the findings to other populations. Future longitudinal studies are warranted to elucidate the causal mechanisms underlying these associations and to inform targeted interventions for depression prevention and management in postmenopausal women.

## 5. Conclusion

The study highlights the importance of considering reproductive history when assessing depression risk in postmenopausal women, suggesting that both early and late childbearing, as well as short birth intervals, may contribute to mental health challenges in this demographic. These findings imply that women with both early and late childbearing, as well as those with closely spaced births, may benefit from enhanced attention to depression screening and support during their postmenopausal years.

## Figures and Tables

**Table 1:** Demographic characteristics of the study participants.

Variables	Overall (*n* = 2375)	Nondepression (*n* = 2104)	Depression (*n* = 271)	*p*-Value
Age (years)	60.10 ± 0.28	60.42 ± 0.30	57.21 ± 0.94	0.002
Race (%)	—	—	—	0.053
Mexican american	208 (8.8%)	187 (7.9%)	21 (0.9%)	—
Other hispanic	251 (10.6%)	213 (9.0%)	38 (1.6%)	—
Nonhispanic black	467 (19.7%)	411 (17.3%)	56 (2.4%)	—
Nonhispanic white	1232 (51.9%)	1097 (46.2%)	135 (5.7%)	—
Other race	217 (9.1%)	196 (8.3%)	21 (0.9%)	—
Family PIR	3.35 ± 0.06	3.47 ± 0.06	2.25 ± 0.14	<0.001
Education level (%)	—	—	—	<0.001
Less than high school	413 (17.4%)	333 (14.0%)	80 (3.4%)	—
High school	347 (14.6%)	299 (12.6%)	48 (2.0%)	—
More than high school	1615 (68.0%)	1472 (62.0%)	143 (6.0%)	—
Marital status (%)	—	—	—	<0.001
Having a partner	1335 (56.2%)	1235 (52.0%)	100 (4.2%)	—
No partner	937 (39.5%)	783 (33.0%)	154 (6.5%)	—
Unmarried	103 (4.3%)	86 (3.6%)	17 (0.7%)	—
Hypertension (%)	—	—	—	<0.001
No	964 (40.6%)	888 (37.4%)	76 (3.2%)	—
Yes	1411 (59.4%)	1216 (51.2%)	195 (8.2%)	—
DM (%)	—	—	—	<0.001
No	1826 (76.9%)	1636 (68.9%)	190 (8.0%)	—
Yes	549 (23.1%)	468 (19.7%)	81 (3.4%)	—
Smoker (%)	—	—	—	<0.001
Never smoke	1412 (59.5%)	1299 (54.7%)	113 ()	—
Former smoke	572 (24.1%)	506 (21.3%)	66 (2.8%)	—
Current smoke	391 (16.5%)	299 (12.6%)	92 (3.9%)	—
Alcohol user (%)	—	—	—	0.002
No	440 (18.5%)	403 (17.0%)	37 (1.6%)	—
Former	424 (17.9%)	359 (15.1%)	65 (2.7%)	—
Mild	851 (35.8%)	774 (32.6%)	77 (3.2%)	—
Moderate	414 (17.4%)	371 (15.6%)	43 (1.8%)	—
Heavy	246 (10.4%)	197 (8.3%)	49 (2.1%)	—
CHD (%)	—	—	—	<0.001
No	2279 (96.0%)	2039 (85.9%)	240 (10.1%)	—
Yes	96 (4.0%)	65 (2.7%)	31 (1.3%)	—
CHF (%)	—	—	—	<0.001
No	2273 (95.7%)	2031 (85.5%)	242 (10.2%)	—
Yes	102 (4.3%)	73 (3.1%)	29 (1.2%)	—
Angina pectoris (%)	—	—	—	<0.001
No	2300 (96.8%)	2058 (86.7%)	242 (10.2%)	—
Yes	75 (3.2%)	46 (1.9%)	29 (1.2%)	—
Heart attack (%)	—	—	—	<0.001
No	2265 (95.4%)	2025 (85.3%)	240 (10.1%)	—
Yes	110 (4.6%)	79 (3.3%)	31 (1.3%)	—
Stroke (%)	—	—	—	0.002
No	2253 (94.9%)	2007 (84.5%)	246 (10.4%)	—
Yes	122 (5.1%)	97 (4.1%)	25 (1.1%)	—
CKD (%)	—	—	—	0.949
No	1848 (77.8%)	1643 (69.2%)	205 (8.6%)	—
Yes	527 (22.2%)	461 (19.4%)	66 (2.8%)	—
Hyperlipidemia (%)	—	—	—	0.089
No	414 (17.4%)	366 (15.4%)	48 (2.0%)	—
Yes	1961 (82.6%)	1738 (73.2%)	223 (9.4%)	—
Oral contraceptive use (%)	—	—	—	0.349
No	694 (29.2%)	625 (26.3%)	69 (2.9%)	—
Yes	1681 (70.8%)	1479 (62.3%)	202 (8.5%)	—
Use female hormones (%)	—	—	—	0.403
No	1470 (61.9%)	1292 (54.4%)	178 (7.5%)	—
Yes	905 (38.1%)	812 (34.2%)	93 (3.9%)	—
Had a hysterectomy (%)	—	—	—	0.009
No	1317 (55.5%)	1197 (50.4%)	120 (5.1%)	—
Yes	1058 (44.5%)	907 (38.2%)	151 (6.4%)	—
Bilateral oophorectomy (%)	—	—	—	0.040
No	694 (29.2%)	625 (26.3%)	69 (2.9%)	—
Yes	1681 (70.8%)	1479 (62.3%)	202 (8.5%)	—
BMI (kg/m^2^)	29.44 ± 0.19	29.28 ± 0.20	30.91 ± 0.78	0.045
Waist circumference (cm)	99.05 ± 0.42	98.67 ± 0.41	102.53 ± 1.77	0.031
Mean energy intake (kcal/day)	1703.93 ± 14.05	1715.63 ± 14.54	1598.10 ± 33.43	<0.001
FBG (mg/dL)	107.03 ± 0.70	105.31 ± 0.62	122.54 ± 3.10	<0.001
TC (mg/dL)	206.95 ± 1.20	206.95 ± 1.11	206.92 ± 4.59	0.995
TG (mg/dL)	124.91 ± 2.16	122.78 ± 2.36	144.22 ± 5.32	<0.001
HDL (mg/dL)	60.92 ± 0.67	61.40 ± 0.74	56.57 ± 1.13	<0.001
BUN (mg/dL)	14.62 ± 0.16	14.69 ± 0.16	13.90 ± 0.49	0.101
UA (mg/dL)	5.01 ± 0.03	5.01 ± 0.04	4.98 ± 0.12	0.797
Scr (mg/dL)	0.82 ± 0.01	0.82 ± 0.01	0.85 ± 0.03	0.195
eGFR (ml/min/1.73 m^2^)	82.91 ± 0.53	82.71 ± 0.56	84.81 ± 1.88	0.289
AFB (years)	23.70 ± 0.17	23.97 ± 0.17	21.20 ± 0.31	<0.001
ALB (years)	27.85 ± 0.15	28.04 ± 0.16	26.17 ± 0.48	<0.001
Birth interval (years)	4.16 ± 0.08	4.97 ± 0.40	4.07 ± 0.08	0.027
Number of pregnancies (times)	2.63 ± 0.03	2.60 ± 0.03	2.89 ± 0.12	0.022
Age at menarche (years)	12.64 ± 0.04	12.67 ± 0.05	12.32 ± 0.13	0.013
Age at menopause (years)	44.69 ± 0.27	45.07 ± 0.28	41.26 ± 0.97	<0.001
Fertile lifespan (years)	32.05 ± 0.28	32.39 ± 0.28	28.93 ± 1.00	<0.001

*Note*: Data are presented as mean ± SD or *n* (%). The categorical variables for baseline characteristics were expressed as unweighted *(n)*, weighted (%).

Abbreviations: AFB, age at first birth; ALB, age at last birth; BMI, body mass index; BUN, blood urea nitrogen; CHD, coronary heart disease; CHF, congestive heart failure; CKD, chronic kidney diseases; DM, diabetes mellitus; eGFR, estimated glomerular filtration rate; Family PIR, family poverty income ratio; FBG, fast blood glucose; HDL-cholesterol, high density lipoprotein-cholesterol; Scr, serum creatinine; TC, total cholesterol; TG, triglycerides; UA, uric acid.

**Table 2:** Associations of AFB, ALB, and birth interval with the prevalence of depression in postmenopausal women.

Reproductive factors	Model 1		Model 2		Model 3	
	OR (95% CI)	*p* for trend	OR (95% CI)	*p* for trend	OR (95% CI)	*p* for trend
AFB	—	<0.001		0.100	—	0.414
≤24	1.00	—	1.00	—	1.00	—
25–27	0.39 (0.24, 0.63)*⁣*^*∗∗∗*^	—	0.62 (0.37, 1.02)	—	0.73 (0.43, 1.23)	—
28–34	0.58 (0.39, 0.85)*⁣*^*∗*^	—	0.83 (0.55, 1.26)	—	0.91 (0.60, 1.40)	—
≥35	0.59 (0.25, 1.39)	—	0.99 (0.39, 2.51)	—	1.17 (0.45, 3.02)	—
ALB	—	0.001	—	0.469	—	0.962
≤24	1.00	—	1.00	—	1.00	—
25–29	0.53 (0.36, 0.78)*⁣*^*∗∗*^	—	0.87 (0.82, 1.58)	—	0.94 (0.61, 1.44)	—
30–34	0.61 (0.39, 0.94)*⁣*^*∗*^	—	0.89 (0.55, 1.44)	—	1.08 (0.65, 1.79)	—
≥35	0.79 (0.58, 1.06)	—	1.14 (0.82, 1.58)	—	1.16 (0.82, 1.63)	—
Birth interval	—	0.049	—	0.130	—	0.078
<3	1.00	—	1.00	—	1.00	—
3	0.99 (0.65, 1.51)	—	0.98 (0.69, 1.41)	—	0.95 (0.65, 1.37)	—
4–6	0.90 (0.62, 1.31)	—	0.89 (0.58, 1.36)	—	0.87 (0.56, 1.34)	—
＞6	0.67 (0.48, 0.95)*⁣*^*∗*^	—	0.71 (0.49, 1.02)	—	0.67 (0.46, 0.98)*⁣*^*∗*^	—

*Note*: Model 1: age and race/ethnicity. Model 2: Model 1 variables plus education level, marital status, family poverty-income ratio, hypertension, diabetes mellitus, smoker, alcohol user; Model 3 was adjusted for Model 2 variables plus body mass index, waist circumference, coronary heart disease, congestive heart failure, angina pectoris, heart attack, stroke, hyperlipidemia, chronic kidney diseases, mean energy intake, oral contraceptive use, use female hormones, had a hysterectomy, bilateral oophorectomy, fast blood glucose, blood urea nitrogen, uric acid, serum creatinine, estimated glomerular filtration rate, total cholesterol, triglyceride, high-density lipoprotein-cholesterol, age at menarche, age at menopause, and fertile lifespan.

Abbreviations: AFB, age at first birth; ALB, age at last birth; CI, confidence interval; OR, odd ratio.

*⁣* ^*∗*^ *p* < 0.05.

*⁣* ^*∗∗*^ *p* < 0.01.

*⁣* ^*∗∗∗*^ *p* < 0.001.

## Data Availability

The data that support the findings of this study are available from the corresponding author upon reasonable request.

## Supporting Information

Supporting Information 1 Supporting Information Figure S1: The RCS curve of the association of AFB with (A) race, (B) family PIR, (C) education level, (D) hysterectomy, (E) bilateral oophorectomy, and (F) oral contraceptive use.

Supporting Information 2 Supporting Information Figure S2: The RCS curve of the association of ALB with (A) race, (B) family PIR, (C) education level, (D) hysterectomy, (E) bilateral oophorectomy, and (F) oral contraceptive use.

Supporting Information 3 Supporting Information Figure S3: The RCS curve of the association of birth interval with (A) race, (B) family PIR, (C) education level, (D) hysterectomy, (E) bilateral oophorectomy, and (F) oral contraceptive use.

Supporting Information 4 Supporting Information Table S1: Subgroups analysis for the associations of AFB with the prevalence of depression in postmenopausal women.

Supporting Information 5 Supporting Information Table S2: Subgroups analysis for the associations of ALB with the prevalence of depression in postmenopausal women.

Supporting Information 6 Supporting Information Table S3: Subgroups analysis for the associations of birth interval with the prevalence of depression in postmenopausal women.
