# Supplementary figures and images for: Associations of Birth Interval With Prevalence of Depression in Postmenopausal Women
Source: Depress Anxiety. 2025 Apr 15;2025:8066072. doi: 10.1155/da/8066072 (PMC12014259; doi:10.1155/da/8066072)

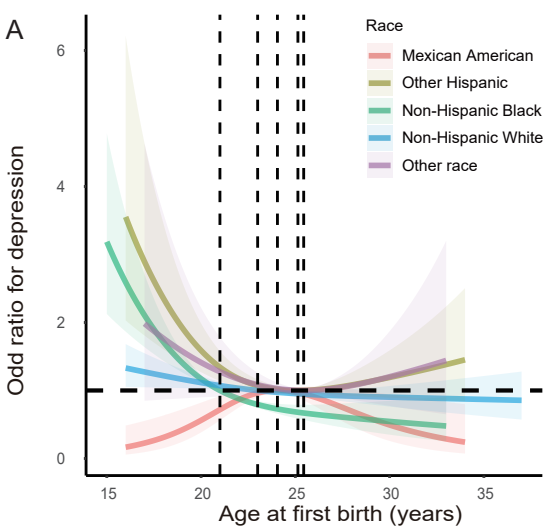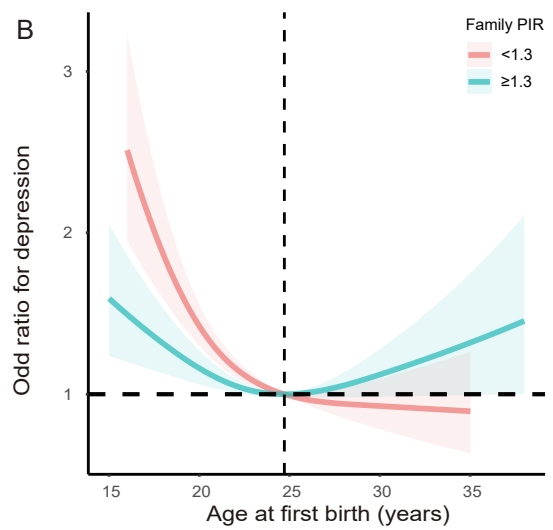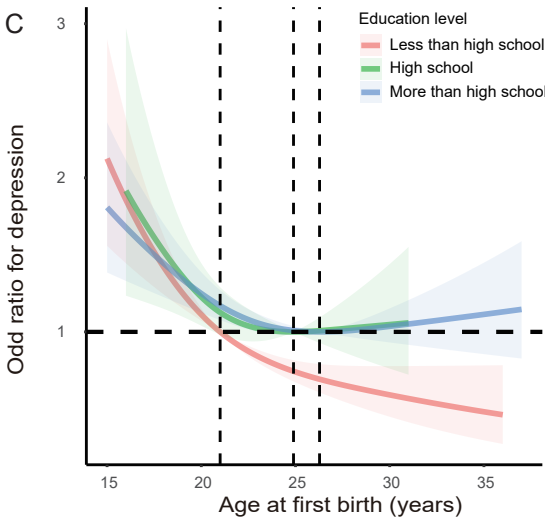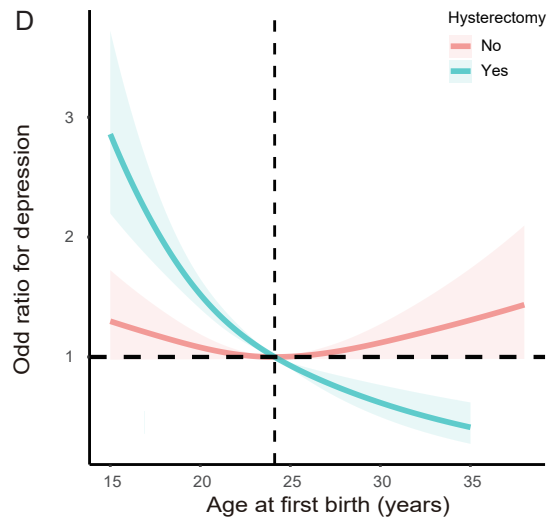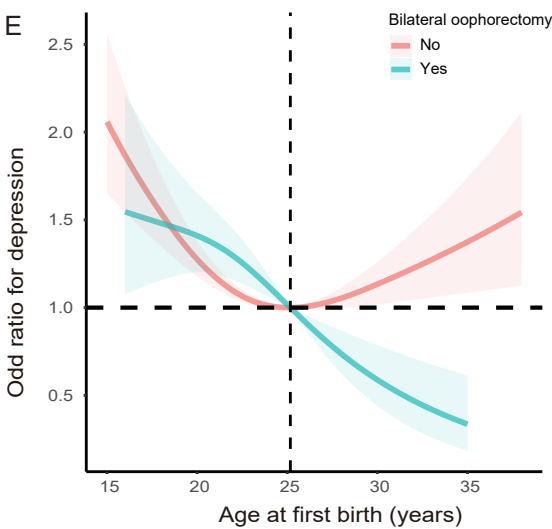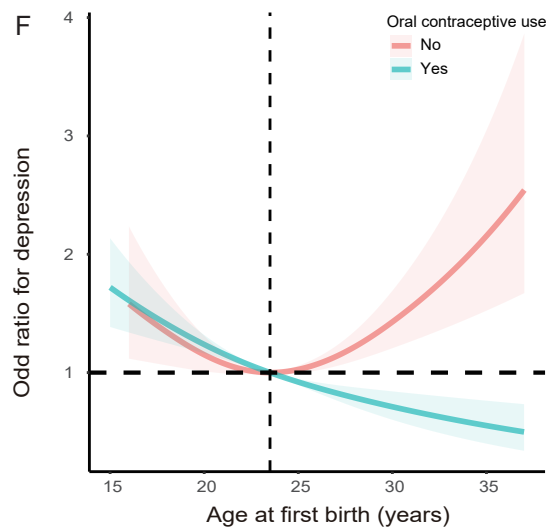

Supplement: Supporting Information 1 — Supporting Information Figure S1: The RCS curve of the association of AFB with (A) race, (B) family PIR, (C) education level, (D) hysterectomy, (E) bilateral oophorectomy, and (F) oral contraceptive use. [file 8066072.f1.pdf]

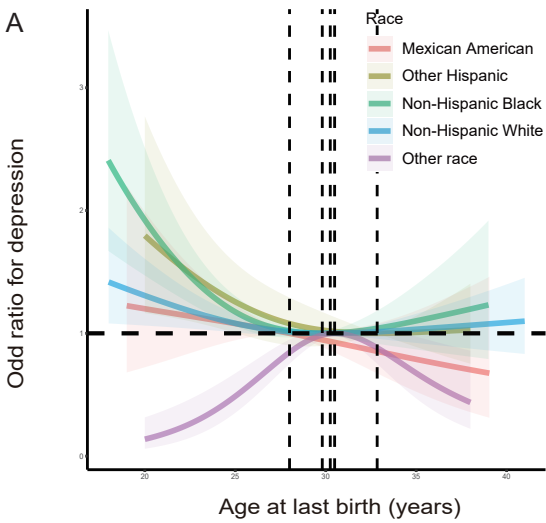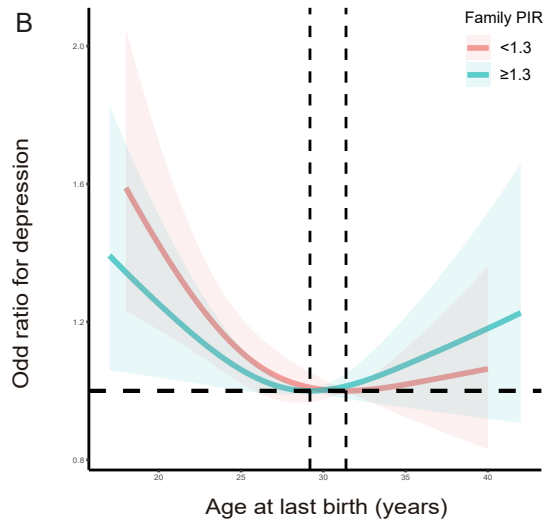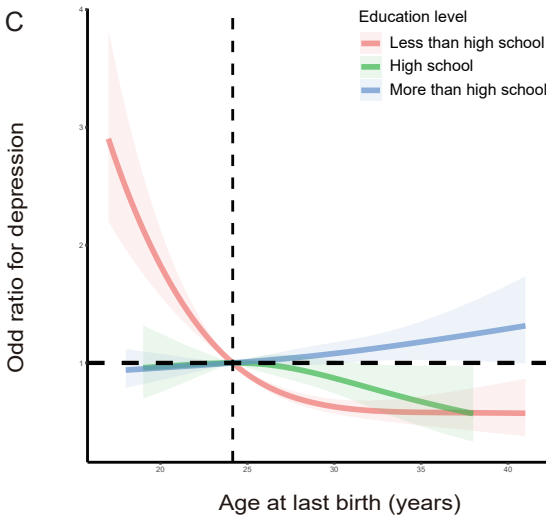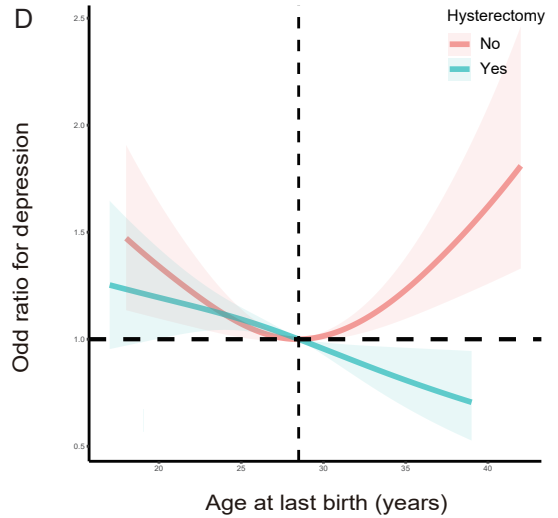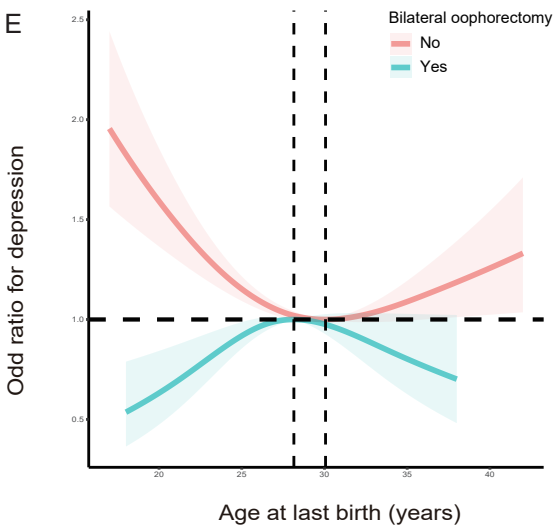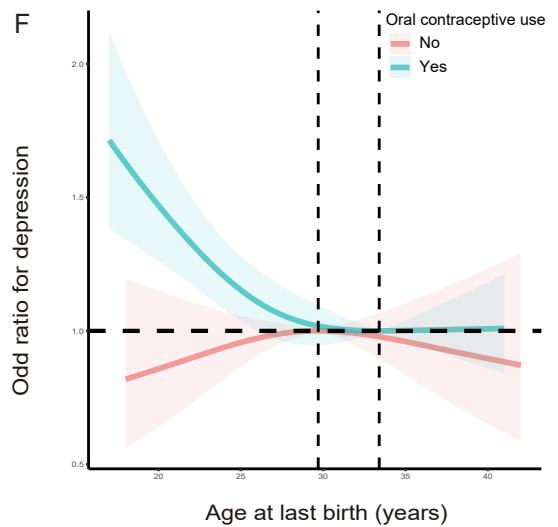

Supplement: Supporting Information 2 — Supporting Information Figure S2: The RCS curve of the association of ALB with (A) race, (B) family PIR, (C) education level, (D) hysterectomy, (E) bilateral oophorectomy, and (F) oral contraceptive use. [file 8066072.f2.pdf]

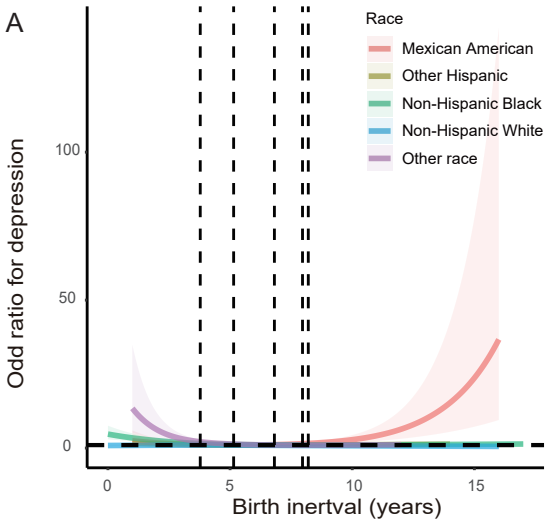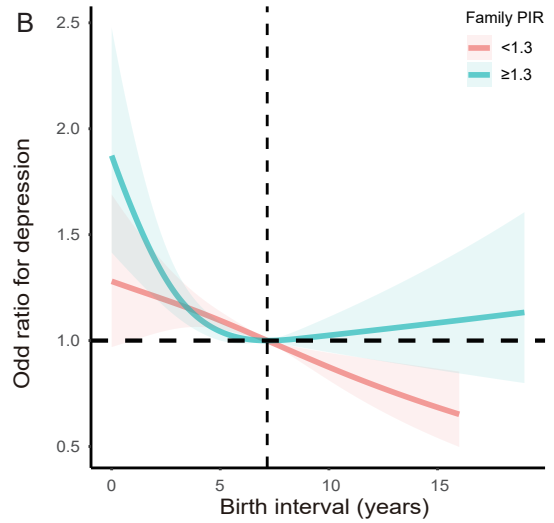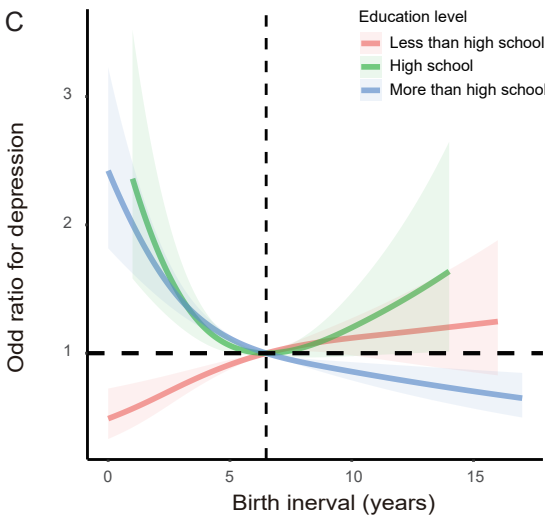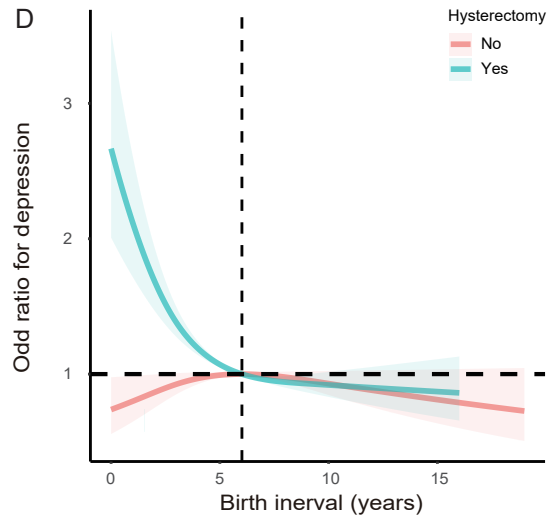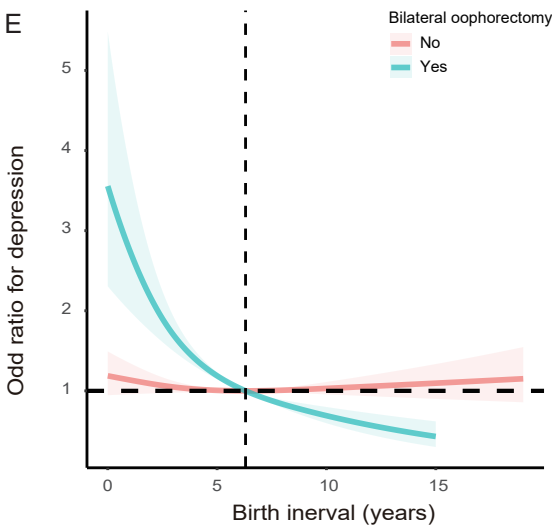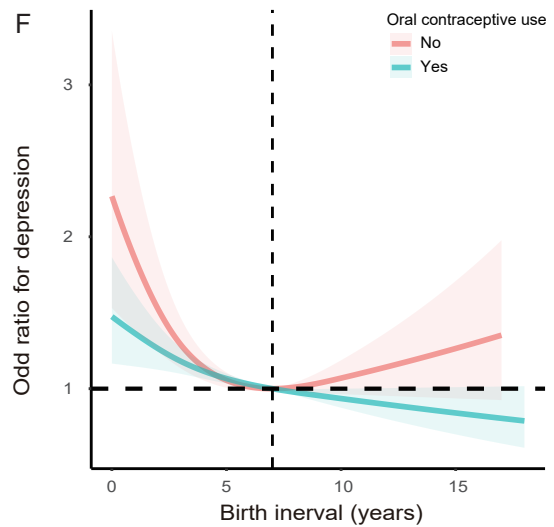

Supplement: Supporting Information 3 — Supporting Information Figure S3: The RCS curve of the association of birth interval with (A) race, (B) family PIR, (C) education level, (D) hysterectomy, (E) bilateral oophorectomy, and (F) oral contraceptive use. [file 8066072.f3.pdf]
