# Supplementary material for: Associations of Birth Interval With Prevalence of Depression in Postmenopausal Women
Source: Depress Anxiety. 2025 Apr 15;2025:8066072. doi: 10.1155/da/8066072 (PMC12014259; doi:10.1155/da/8066072)
Supplement: Supporting Information 5 — Supporting Information Table S2: Subgroups analysis for the associations of ALB with the prevalence of depression in postmenopausal women. [file 8066072.f5.docx]

| **Supplementary Table 2.** Subgroups analysis for the associations of ALB with the prevalence of depression in postmenopausal women. | | | | | | |
| --- | --- | --- | --- | --- | --- | --- |
|  | ≤24 | 25-29 | 30-35 | ≥35 | *P* for trend | *P* for interaction |
|  | OR (95%CI) | OR (95%CI) | OR (95%CI) | OR (95%CI) |  |  |
| Race |  |  |  |  |  | <0.001 |
| Mexican American | 1.00 | 0.98 (0.28, 1.82) | 0.84 (0.45, 1.34) | 0.42 (0.06, 1.01) | 0.732 | |
| Other Hispanic | 1.00 | 0.94 (0.27, 2.08) | 0.51 (0.12, 2.18) | 1.20 (0.28, 5.27) | 0.695 |  |
| Non-Hispanic Black | 1.00 | 0.89 (0.39, 2.00) | 0.61 (0.16, 2.35) | 0.77 (0.30, 2.01) | 0.415 |  |
| Non-Hispanic White | 1.00 | 0.94 (0.49, 1.53) | 0.73 (0.39, 1.39) | 1.16 (0.72, 1.88) | 0.610 |  |
| Other race | 1.00 | 3.89 (0.45, 5.55) | 1.51 (0.20, 2.55) | 1.46 (0.15, 2.43) | 0.569 |  |
| Family PIR |  |  |  |  |  | 0.155 |
| <1.3 | 1.00 | 0.97 (0.53, 1.58) | 0.80 (0.40, 1.25) | 1.54 (0.92, 2.59) | 0.862 |  |
| ≥1.3 | 1.00 | 0.99 (0.63, 1.56) | 0.85 (0.43, 1.69) | 0.93 (0.54, 1.59) | 0.626 |  |
| Education level |  |  |  |  |  | 0.002 |
| Less than high school | 1.00 | 0.66 (0.34, 1.31) | 0.14 (0.03, 0.63) * | 0.48 (0.20, 1.17) | 0.016 |  |
| High school | 1.00 | 1.56 (0.70, 3.51) | 1.12 (0.37, 3.40) | 0.77 (0.14, 4.29) | 0.966 |  |
| More than high school | 1.00 | 1.26 (0.74, 2.15) | 1.29 (0.67, 2.50) | 1.45 (0.89, 2.35) | 0.432 |  |
| Hysterectomy |  |  |  |  |  | 0.063 |
| No | 1.00 | 0.94 (0.57, 1.86) | 1.16 (0.64, 2.10) | 1.34 (0.71, 2.52) | 0.384 |  |
| Yes | 1.00 | 0.89 (0.63, 1.61) | 0.74 (0.39, 1.42) | 0.61 (0.24, 1.54) | 0.295 |  |
| Bilateral oophorectomy |  |  |  |  |  | 0.059 |
| No | 1.00 | 0.98 (0.66, 1.46) | 0.86 (0.54, 1.39) | 0.87 (0.50, 1.50) | 0.493 |  |
| Yes | 1.00 | 2.19 (1.07, 4.49) * | 1.39 (0.44, 4.37) | 1.02 (0.39, 2.65) | 0.684 |  |
| Oral contraceptive use |  |  |  |  |  | 0.087 |
| No | 1.00 | 1.24 (0.60, 2.55) | 1.58 (0.68, 3.69) | 1.16 (0.48, 2.77) | 0.556 |  |
| Yes | 1.00 | 0.74 (0.45, 1.22) | 0.83 (0.45, 1.53) | 0.83 (0.43, 1.42) | 0.289 |  |

Abbreviations: ALB, age at last birth; Family PIR, family poverty income ratio; **P* < 0.05; OR, odd ratio; CI, confidence interval. Analyses was adjusted for age, race/ethnicity, education level, marital status, family poverty-income ratio, hypertension, diabetes mellitus, smoker, alcohol user, body mass index, waist circumference, coronary heart disease, congestive heart failure, angina pectoris, heart attack, stroke, hyperlipidemia, chronic kidney diseases, mean energy intake, oral contraceptive use, use female hormones, had a hysterectomy, bilateral oophorectomy, fast blood glucose, blood urea nitrogen, uric acid, serum creatinine, estimated glomerular filtration rate, total cholesterol, triglyceride, high-density lipoprotein-cholesterol, age at menarche, age at menopause, and fertile lifespan. All *P*-values were calculated using ≤24 as the reference.
